# Primary visual cortex BOLD responses to relative localization of sounds at 7T

**DOI:** 10.1016/j.isci.2026.116075

**Published:** 2026-05-25

**Authors:** M. Riberto, M.B. Amadeo, A. Inuggi, M. Costagli, C. Campus, M. Gori, M.C. Morrone

**Affiliations:** 1 Unit for Visually Impaired People, Italian Institute of Technology, Genoa, Italy; 2 IRCCS Ospedale Policlinico San Martino, Genoa, Italy; 3 Department of Neuroscience, Rehabilitation, Ophthalmology, Genetics, Maternal and Child Health, University of Genoa, Genoa, Italy; 4 Department of Translational Research on New Technologies in Medicine and Surgery, University of Pisa, Pisa, Italy

**Keywords:** neuroscience, sensory neuroscience, cognitive neuroscience

## Abstract

To clarify the computational role and dynamics of auditory-driven cross-modal activations in visual cortices, we investigated the recruitment of the primary visual cortex (V1) during auditory spatial perception using fMRI and dynamic causal modeling (DCM). Ten participants estimated the relative position of sounds with respect to two spatial landmarks. We found significant activation of V1 in response to the sound to localize, along with bilateral activation in the primary auditory cortex (A1) and intraparietal sulcus (IPS; pFWE < 0.001). Moreover, we observed task-dependent changes in the effective connectivity between V1, A1, and IPS: in particular, the coupling between A1 to IPS, as well as from IPS to V1 increased positively. This study suggests that judgments of the relative distance between sounds elicit activity in V1, and V1 recruitment is likely explained by an increased effective connectivity from IPS. This network may support the brain ability to perform high-level spatial computations in the auditory domain.

## Highlights

• V1 is activated during a purely acoustic spatial localization task
• This is explained by increased effective connectivity with the intraparietal sulcus
• This network may support high-level auditory spatial computations

## Graphical abstract

**Figure:**
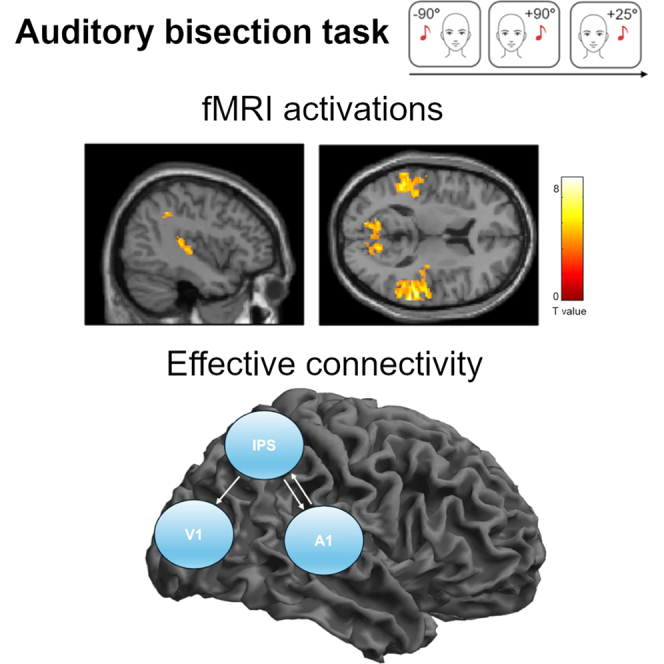

## Introduction

To date it is well-known that primary sensory cortices receive information from other senses through cortical feedback and top-down pathways.^1^ Primary visual cortex (V1) is interconnected with non-visual areas and can respond even in the absence of visual stimulation.^2^^,^^3^ This phenomenon has been extensively demonstrated in rodents^4^^,^^5^ and cats,^6^ where single neuronal responses are modulated by auditory signals. Auditory cross-modal activations have been observed in humans too.^7^

In both animals and humans, the auditory activations in V1 are putatively mediated by direct anatomical connections between the primary auditory cortices (A1) and V1.^8^^,^^9^^,^^10^^,^^11^ However, the circumstances under which these circuits are active, and their functional significance are still debated. Recent theories suggest that visual cortex supports non-visual functions,^12^ such as higher-level representations of semantic content of sounds, sound imagery,^13^ and spoken language.^14^ Recent electrophysiological evidence indicates a specific involvement of visual cortices in processing spatial information even for auditory inputs^15^^,^^16^: the V1 high definition of the retinotopic map may be used to decode spatial position of auditory sources. However, other evidence in rodents supports the view that V1 activity to sound may be an unspecific response to arousal or to attentional allocation.^17^

To clarify the computational role and the dynamics of cross-modal activations of the visual regions, we used ultra-high field functional magnetic resonance imaging (fMRI) to explore the recruitment of V1 during auditory space perception, and dynamic causal modeling (DCM) to investigate context-sensitive changes in neuronal coupling between V1, A1, and the intraparietal sulcus (IPS), an associative region involved in cross-modal integrative processing of spatial information.^18^^,^^19^^,^^20^^,^^21^^,^^22^ In light of the role of vision in cross-modal spatial perception^23^ and the superior spatial acuity of V1,^24^ we expect that V1 responds during a purely auditory task involving relative distance judgments between two sounds. This may suggest that V1 is recruited when a construction of complex spatial metrics is necessary, supporting the brain’s ability to perform intricate auditory spatial computations.

## Results

**Figure 1:**
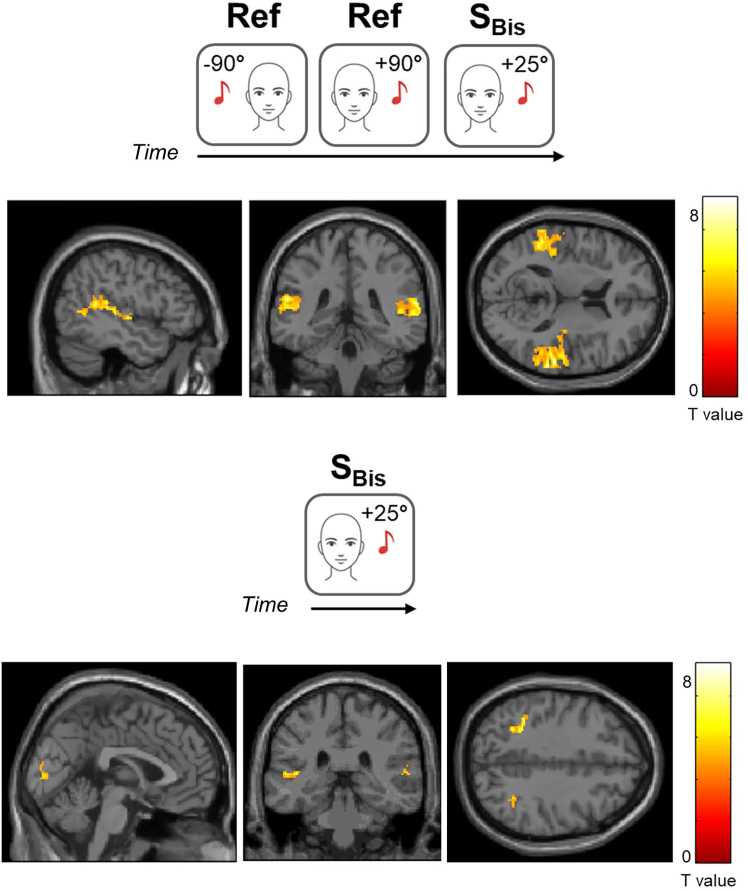
fMRI results Here, we show the BOLD activations in response to sounds during the bisection task. (Top) Whole-brain activations in the bilateral A1 and IPS in response to Ref and S_Bis_. (Bottom) S_Bis_ significantly activated the bilateral V1, A1, and IPS. These results were cluster-extent based thresholded for multiple comparisons correction (pFWE < 0.05). The color bar represents *t* values associated with each contrast. Abbreviations, Ref, reference landmarks presented at ±90° from the midline; S_Bis_, sound presented at ±25° from the midline in the bisection task; A1, primary auditory cortex; IPS, intraparietal sulcus; V1, primary visual cortex.

Ten participants performed a spatial bisection task. They had to judge a sound relative spatial position (S_Bis_; ±25°) with respect to two landmarks (Ref; ±90°) in a 7T scanner. The auditory stimuli (i.e., Ref and S_Bis_) significantly activated the bilateral auditory cortex (Figure 1), both in the whole brain analysis and in the region of interest (ROI) analysis limited to A1. Also, the bilateral IPS and the right V1 were significantly activated by sounds. No significant clusters showed reduced activation during the task. Comparing the blood oxygenation level-dependent (BOLD) response during the bisection task against the baseline (i.e., S_Bis_ > 0), we observed bilateral V1 activations in line with our hypothesis. We also found that bilateral A1 and IPS were significantly activated by S_Bis_, with no negative BOLD responses (Figure 1). The statistics and the significance of these results are presented in Table 1. For completeness, we explored V1 activations associated with the spatial reference stimuli (Ref) only and we found no significant BOLD responses (Figure S1).

**Table 1:** fMRI results

Contrast	Analysis	Brain region	Coordinates	k	*t*	pFWE
**Positive effect of the bisection task**	Whole brain	A1 left	−56 –38 18	756	7.73	<0.001
		A1 right	62 –30 14	1,075	7.99	<0.001
		IPS left	−38 –44 40	50	5.30	0.01
		IPS right	30 –44 34	48	4.09	0.02
		V1 right	12 –68 12	195	5.38	<0.001
	ROIs	A1 left	−50 –34 14	292	6.89	<0.001
		A1 right	52 –28 8	267	6.24	<0.001
**S**_**Bis**_**>0**	Whole brain	A1 left	−44 –30 8	85	9.16	<0.001
		A1 right	54 –30 8	25	5.30	0.04
		Ips left	−38 –44 40	89	7.10	<0.001
		Ips right	32 –54 40	26	5.58	0.04
		V1 left	−12 -80 12	48	6.95	0.001
		V1 right	2 –86 6	32	6.86	0.01
	ROIs	V1 left	−12 –80 12	48	6.95	0.001
		V1 right	2 –86 6	32	6.86	0.01

Coordinates (center of the cluster), size in voxels (k) and the statistics of brain regions significantly activated in each group analysis.

A1, primary auditory cortex; IPS, intraparietal sulcus; V1 primary visual cortex.

For exploratory purposes, we conducted an auditory localization task after the time series with the spatial bisection task to explore whether the two tasks involve different brain regions. Specifically, we asked participants to mentally assess whether a sound (S_Loc_; ±25°) was coming from either the right or left sides with eyes closed (see Figure S2). We observed activations in the bilateral A1 and right precuneus associated with S_Loc_ (i.e., S_Loc_ > 0). In addition, the bilateral V1 was more activated by S_Bis_ along with the left IPS (S_Bis_ > S_Loc_). These results are summarized in Table S1 and Figure S3.

**Figure 2:**
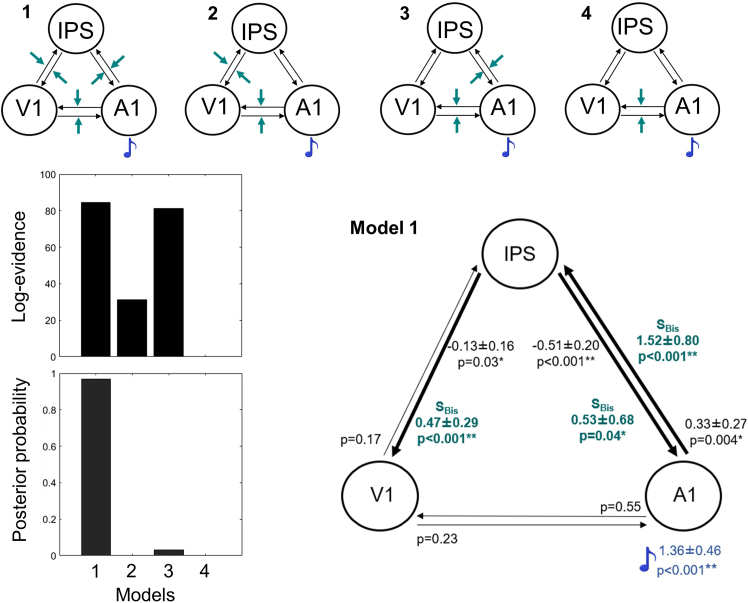
DCM results (Top) Four candidate DCMs were compared using fixed-effect BMS, differing in changes in effective connectivity due to S_Bis_ (displayed in green color). Specifically, in model 1, the full connected model, we predicted that the task modulated the effective connectivity between low-level regions (V1 and A1) and between them and the IPS. In model 2–3, we expected a modulatory effect of task on the coupling between V1 and A1, and between IPS and V1 (model 2), and between IPS and A1 (model 3). Finally, in model 4, we expected a modulatory effect of task on the connectivity between V1 and A1. (Bottom) Fixed-effect BMS showed that model 1 is the optimal model. We estimated the parameters of the connectivity in model 1 and entered each of them (i.e., *baseline* connectivity, black arrows and black font; S_Bis_ driving input in A1, blue font; modulatory effect of S_Bis_, green font) as dependent variables in one-sample *t* tests, testing the null hypothesis that each parameter had mean equal to 0. ∗ *p* < 0.05, ∗∗ *p* < 0.001. Abbreviations, S_Bis_, sound presented at ±25° from the midline in the bisection task; A1, primary auditory cortex; IPS, intraparietal sulcus; V1, primary visual cortex.

Having established the foci of activation in the bisection task, we investigated the putative circuitry engaged during the bisection task (S_Bis_) using DCM,^25^ using all possible configurations between the three nodes. As shown in Figure 2 (bar graphs on the left), the full connected model (M1) is the best model according to the fixed-effect Bayesian model selection (BMS) analysis with the highest posterior probability (M1 = 0.97). Specifically, the one-sample *t* tests of M1 estimated parameters revealed a positive *baseline* connectivity from A1 to IPS (0.33 ± 0.27, t = 3.89, *p* = 0.004), reflecting an excitatory effect of A1 on IPS. Conversely, we observed a negative *baseline* connectivity from IPS to A1 (−0.51 ± 0.20, t = −7.87, *p* < 0.001) and to V1 (−0.13 ± 0.16, t = −2.58, *p* = 0.03), that may indicate an inhibitory effect of IPS on both A1 and V1. In addition, we observed that S_Bis_ induced a positive activation in A1 (1.36 ± 0.46, t = 9.31, *p* < 0.001). We explored the task-dependent changes in effective connectivity using only S_Bis_ that produced positive activity in all three ROIs: in particular, the bisection task modulated the connectivity from A1 to IPS (1.52 ± 0.80, t = 5.99, *p* < 0.001) and from IPS to A1 (0.53 ± 0.68, t = 2.46, *p* = 0.04), by increasing positively the coupling between these areas. Finally, S_Bis_ also enhanced the connectivity from IPS to V1 (0.47 ± 0.29, t = 5.04, *p* < 0.001). The DCM results show that the bisection task relies on a complex circuit, which implies enhanced effective connectivity not only between A1 and IPS as expected,^26^^,^^27^ but also from IPS to V1. This circuit may support the higher cross-modal activation of V1 reported with fMRI analyses during the bisection task.

## Discussion

Results demonstrate that V1 responds during a purely auditory task involving relative distance judgments between two sounds. V1 activation in response to sounds is accompanied by an increased effective connectivity from IPS to V1.

V1 recruitment during the bisection task is coherent with a growing body of research highlighting a complementary role of vision in improving spatial localization when auditory input alone may be less precise,^23^ given the superior spatial localization acuity of V1.^24^ This view is also consistent with the lack of identified topographic maps of space in A1^28^^,^^29^ and with evidence indicating that auditory spatial localization can also be achieved via opponent population coding.^30^^,^^31^ By showing a cross-modal recruitment of V1 during the bisection task, our findings might suggest that visual occipital regions are recruited when humans need to perform complex auditory spatial operations. We speculate that the involvement of visual areas may serve a complementary and compensatory role, particularly under conditions of increased spatial uncertainty or high task demands. However, further research is required to confirm this hypothesis, likely involving a control experiment with either a more challenging (e.g., sounds varying in elevation) or simpler (e.g., no relative judgments) task.

In our study, the sound (S_Bis_) position needs to be estimated with respect to external landmarks. This task is likely more complex than simple binary localization relative to the participant’s internal midline, as it requires integrating the relative spatial distances among the stimuli, rather than relying solely on their absolute positions or presentation order. In order to isolate and extract the response to the reference stimuli (Ref), it was necessary to present them well before the stimulus to localize (S_Bis_). This imposed the requirement for the participant to use a memory representation, increasing further the complexity of the task. While we cannot distinguish whether V1 activity is related to the increased memory load of the task or to a general processing of relative metrics between different spatial locations, the fact that the discrimination location accuracy and precision are the same with and without reference stimuli^32^ would support the second explanation. Consistently with this idea, Zimmer and colleagues^33^ showed that activation in V1 was lacking during sound lateralization but appeared when participants had to localize sounds in combination with gaze directed to the right and left side. They concluded that activity in visual cortices is implicated in the transformation of auditory spatial coordinates responsible for maintaining the perceptual alignment of audition and vision during changes in gaze direction. Our results, collected with eyes closed, add that visual cortex activity may be necessary to perform manipulations of auditory spatial coordinates, independently of the visual input.

While showing a lack of topographic projection from A1 to V1, a recent study in mice^34^ suggests that V1 is not engaged in the detection of low-level acoustic features, such as sound localization, but it uses the varied and widespread spatial information provided by A1 for higher-level analyses of space perception. These results support the view that V1 responses to sound may compute the relative positions of sounds to integrate them with visual information. This is in contrast with the evidence from Bimbard et al.,^17^ that consider the cross-modal response in V1 a mere result of increased arousal elicited by the sound. Our data in humans cannot discriminate between these two interpretations, given that the reference stimuli might have increased arousal, attention and memory load. However, the fact that V1’s involvement is mediated by the activity of the IPS, an area deeply involved in multisensory spatial representation and attention,^18^^,^^19^^,^^20^^,^^21^^,^^22^ potentially may call for a role of V1 in high cognitive auditory spatial tasks. The recruitment of IPS during the bisection task, which requires relative spatial estimations, aligns with a recent study reporting maximal proportion of allocentric-selective channels in the IPS of epilepsy patients.^35^

We instructed participants to perform the task with eyes closed to exclude any visual input to V1 that could affect our analyses. Indeed, it is well known that the BOLD response is affected by oculomotor responses even when eyes are closed.^36^^,^^37^ To overcome this issue, in our previous works with electroencephalography,^38^^,^^39^ participants performed the bisection task with their gaze fixed, and we monitored eye movements using electrooculogram. Ruling out spurious eye movements toward the apparent sound location, we showed an event-related potential (ERP) response to sound in V1. Nevertheless, we acknowledge oculomotor activity as a potential confound in the current study and the need for follow-up studies to investigate it, especially for small drift eye-movements that are difficult to measure.

Our results provide important insights for understanding cross-modal pathways and multisensory processes in the brain, and for studying sensory deficits. For instance, blind individuals struggle with relative localization of sounds,^32^^,^^40^ possibly due to a reduced coupling from associative parietal areas to V1. In blind individuals, in whom we demonstrated a deficit in bisection performance,^32^ we might expect an alteration of the average effective connectivity from IPS to V1.

To conclude, this study suggests that judgment of the relative distance between sounds requires the recruitment of V1, which is explained by an increased effective connectivity from IPS to V1. We speculate that this network may support the brain's ability to compute the relative positions of sounds and could inform further research into brain plasticity in the absence of vision, inspiring rehabilitation approaches for visual loss.

### Limitations of the study

Our study presents several limitations that can be addressed in future works. First, our sample size was relatively small, which also limited the possibility of examining sex effects on brain activations; we opted for this considering the resource constraints and the high number of trials per participant, which ensured an adequate signal-to-noise ratio. Moreover, this study did not include visually impaired participants, which limits the generalizability of our findings. Future research should expand the sample size to replicate these results and compare neural responses between sighted individuals and blind adults. In addition, we did not randomize the order of the spatial tasks across participants, as the localization task was included in a second experimental stage for exploratory purposes. Future studies should randomize the order of spatial tasks to better examine potential task-related modulation of effective connectivity. Research is also needed to further investigate the role of spatial uncertainty or task demands. To keep computational complexity in reasonable limits, the models did not include decision making cortices that in principle could contribut to the task modulation of connectivity. This choice is also related to the nature of the experiment, wherein participants were asked not to make an explicit judgment, but only to mentally represent the position of the sounds. Possibly, by asking participants to produce a response, we could observe the recruitment of prefrontal regions involved in decisionmaking and thus include them in the model. Another caveat is related to the large macro-anatomical variability in the temporal lobe and the different nature of connections between belt and parabelt that might have affected the localization of the activations, with some peaks in voxels not limited to the primary auditory cortex. Finally, the effect of oculomotor activity should be further investigated.

## Resource availability

### Lead contact

Requests for further information and resources should be directed to and will be fulfilled by the lead contact, Monica Gori (monica.gori@iit.it).

### Materials availability

This study did not generate new unique materials.

### Data and code availability

• Data have been deposited at Zenodo and is publicly available as of the date of publication. DOI is listed in the key resources table.
• This study does not report original code.
• Any additional information required to reanalyze the data reported in this study is available from the lead contact upon request.

## Acknowledgments

This research was supported by the 10.13039/501100000780European Union (EU) and 10.13039/501100007601Horizon 2020: grant agreement no. 832813—ERC Advanced “Spatio-temporal mechanisms of generative perception— GenPercept” and grant agreement no. 948349—ERC Starting Grant “The role of vision on perceptual space representation.”

## Author contributions

Conceptualization, M.R., M.B.A., A.I., M.C., C.C., M.G., and M.C.M.; methodology, M.R., M.B.A., A.I., M.C., C.C., and M.C.M.; investigation, M.B.A., A.I., M.C., and C.C.; writing – original draft, M.R., M.B.A., A.I., M.C., C.C., M.G., and M.C.M.; writing – review and editing, M.R., M.B.A., A.I., M.C., C.C., M.G., and M.C.M.; funding acquisition, M.G. and M.C.M.; resources, M.G. and M.C.M.; supervision, C.C., M.G., and M.C.M.

All co-authors have read and approved the final version of the article.

## Declaration of interests

The authors declare no competing interests.

## STAR★Methods

### Key resources table

**Table:** 

REAGENT or RESOURCE	SOURCE	IDENTIFIER
**Deposited data**		

fMRI data	Zenodo	https://doi.org/10.5281/zenodo.19552196

**Software and algorithms**		

MATLAB	The Mathworks	RRID: SCR_001622
SPM 12	Wellcome Trust Center for Neuroimaging, University College London	RRID: SCR_007037
FSL	Oxford Center for Functional MRI of the Brain, University of Oxford	RRID: SCR_002823
Jülich histological atlas	Forschungszentrum Jülich, Institute of Neuroscience and Medicine (INM-1)	RRID: SCR_002308

**Other**		

VisuaStim Digital MR-compatible earphones	Resonance Technology Inc., Los Angeles, USA	
Discovery MR950 7T MRI system	GE Healthcare, Chicago, USA	

### Experimental model and study participant details

#### Participants

Ten healthy participants took part in the study (Right-handed = 9, Female = 6; Age (mean ± standard deviation) = 26.8 ± 3.6 years). Participants were predominantly of European descent (*n* = 9), with one participant of Asian descent (*n* = 1). Race and ethnicity were not separately assessed. All participants resided in Italy at the time of the study (for participant demographics see Table S2). All participants had normal or corrected-to-normal vision. They provided written informed consent prior to the experiment and were reimbursed for their participation. Experimental procedures are in line with the Declaration of Helsinki and were approved by the regional ethics committee (Comitato Etico Pediatrico Regionale—Azienda Ospedaliero-Universitaria Meyer—Firenze (FI)) and by the Italian Ministry of Health, under the protocol ‘Plasticità e multimodalità delle prime aree visive: studio in risonanza magnetica a campo ultra-alto (7T)’ version #1 dated 11/11/2015 (protocol number: 0001650-15/01/2016-DGDMF-COD_UO-P).

### Method details

#### Materials

**Figure 3:**
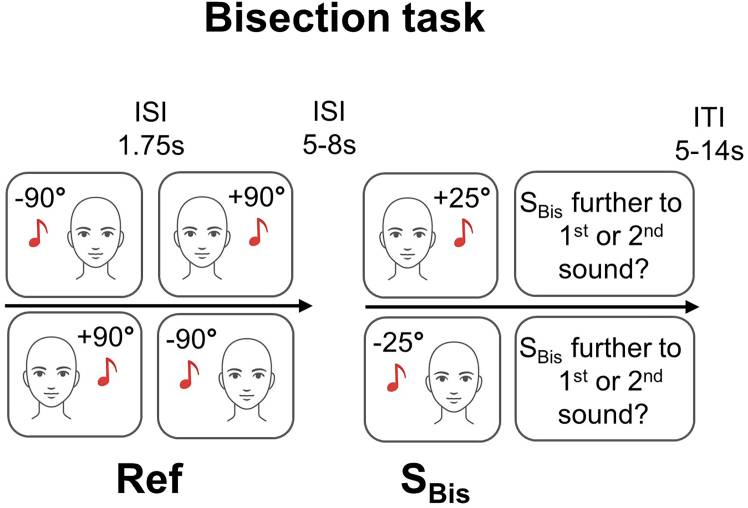
Experimental procedure During fMRI, ten participants performed an auditory spatial bisection task. Their task was to mentally assess which one of the two landmarks (Ref) at ± 90° from midline was spatially further away from the S_Bis_ placed at ± 25° from midline. Abbreviations, ISI, inter-stimulus interval; ITI, inter-trial interval.

The stimulus set comprises 180 sounds (white noise) with a duration of 600 ms each (see Figure 3). During the spatial bisection task, 60 sounds were presented at ± 25° from body midline (S_Bis_: 30 at −25° and 30 at +25°), and 120 sounds were presented in pairs at ± 90° from body midline (ref. 60 at −90° and 60 at +90°). S_Bis_ was always preceded by the two Ref at a temporal separation of 1.75s. During the spatial localization task, each sound (S_Loc_, 60 in total: 30 at −25° and 30 at +25°) was presented without any landmark (see Figure S2).

#### Experimental procedure

Each participant underwent one 7T MRI exam, including both anatomical and functional examinations, as detailed in the following subsection. During functional scanning, participants performed an auditory spatial bisection task (see Figure 3). During the spatial bisection task, participants were asked to mentally estimate the relative spatial position of S_Bis_ with respect to the position of two landmark sounds; the order of the two landmark sounds was randomly assigned through trials presented 5–8 s before S_Bis_. The bisection task was to mentally assess which one of the two landmark sounds was spatially further away from S_Bis_. The task has been previously used to explore early recruitment of occipital areas with electroencephalogram (EEG).^38^ The bisection task had 60 trials in total with an inter-trial interval (ITI) between 5 and 14 s.

After collecting bisection task data, we added on the same participant two series of localization task, wherein participants had to assess whether a stimulus placed at ± 25° from midline (S_Loc_) was coming from either the right or left sides with eyes closed. The localization task had the same number of trials and the ITI of the bisection task. It was conducted as part of the original experimental design and was embedded in the overall structure of the experiment and planned analyses to explore whether the two tasks involve different brain regions. Although its findings can be considered only as exploratory, we included them in the manuscript in the interest of transparency. We did not randomize the order of the task; thus, the limitation is that we cannot decouple the order and the task effects. The total duration of the fMRI experiment was approximately 40 min, divided into 5 series (1–3: bisection task; 4–5: localization task), with short breaks of a few seconds in between. Stimuli were prepared in MATLAB (Mathworks, Natick, USA) and administered to the participants via MR-compatible earphones (VisuaStimDigital, Resonance Technologies, Los Angeles, USA) during fMRI acquisition.

Images were acquired with a Discovery MR950 7T MRI system (GE Healthcare, Chicago, USA) equipped with a two-channel transmitter and 32-channel receiver head coil (Nova Medical, Wilmington, USA) and a gradient system with maximum amplitude = 50 mT/m and slew rate = 200 mT/m/ms. Functional images were acquired with a two-dimensional gradient-echo EPI sequence with 34 slices with thickness 1.9 mm and slice spacing = 0.1 mm, Field of View (FOV) = 192 mm, matrix size = 96, resulting in a spatial resolution of 2 × 2 mm^2^ in-plane, covering the early visual and auditory cortices, as well as the intraparietal sulcus, Time of Repetition (TR) = 1750 ms, Time of Echo (TE) = 21 ms, flip angle (FA) = 76°, receiver bandwidth = ±250 kHz, ASSET acceleration factor = 2, phase encoding direction: Anterior-to-Posterior. No resampling was performed during the reconstruction. One additional acquisition of ten volumes, with exactly same parameters but for the reversed phase encoding direction (Posterior-to-Anterior) was used for geometrical distortion correction. The acquisition protocol included also whole-brain anatomical T1-weighted imaging with magnetization-prepared rapid acquisition of gradient echo sequence with TR = 5.3 ms, time of inversion = 600 ms, TE = 2.4 ms, FA = 7°, with FOV = 256 mm, matrix size = 320, resulting in an isotropic spatial resolution of 0.8 × 0.8 × 0.8 mm^3^ and scan duration of 4:24.

### Quantification and statistical analyses

#### fMRI

##### Preprocessing

The distortions caused by magnetic field inhomogeneities were calculated using the EPI images acquired with opposite phase encoding directions using the following FSL commands: *topup*, which calculates the susceptibility-induced off-resonance field, and *applytopup* which applies it to original images.^41^^,^^42^ Distortion-corrected EPI images then underwent the conventional Statistical Parametric Mapping (SPM12)^43^ preprocessing pipeline: a rigid body motion correction, slice timing correction, co-registration with the whole-brain T1 image, normalization to the Montréal Neurological Institute (MNI) stereotaxic space and smoothing with an isotropic Gaussian kernel of 4 mm full-width-at-half-maximum.

##### First-level analyses

For each participant, pre-processed functional data from each voxel were analyzed using a general linear model (GLM) approach. Stimuli within each task were modeled as separate conditions, beginning with each stimulus presentation onset, using the canonical hemodynamic stimulus response function in SPM12, and included in the model as regressors of interest. Specifically, three regressors were entered in the GLM for each participant: the reference stimuli (Refs) and the target stimulus (S_Bis_) for the spatial bisection task, and the stimulus (S_Loc_) for the spatial localization task, along with six motion correction parameters as regressors of no interest. We used the regressors associated with Ref and S_Bis_ for the main analyses, and S_Loc_ only for exploratory purposes. High temporal filtering with cutoff value of 128 s was also applied. From this GLM analysis, we obtained a single beta image for each regressor. Then, we generated t contrast images for each regressor against the baseline to investigate the positive effect of the bisection task, and one contrast image testing higher activation in the V1 for S_Bis_ for each participant. Despite the individual sounds at ±25° from the midline being the same in the two tasks (although embedded in different stimulus contexts), we expected A1 recruitment also for S_Loc_, with a selective activation of V1 during S_Bis_. To test these hypotheses, we generated one t contrast image for S_Loc_ against the baseline to investigate the positive effect of the localization task, and one contrast image testing higher activation in the V1 for S_Bis_ against S_Loc_ (i.e., S_Bis_ > S_Loc_) for each participant.

##### Second-level analyses

First, we entered the contrasted images for the positive effect of the bisection task (i.e., Ref and S_Bis_) from each participant as dependent variables in a paired *t* test with task conditions as a within-participant factor for manipulation check, and the positive effect of S_Bis_ (S_Bis_ > 0) as a dependent variable in a one-sample *t* test. We also tested the opposite contrasts to exclude the presence of negative significant activations. The analyses testing the positive effect of the task and the experimental hypothesis were performed both whole-brain and in Regions of Interest (ROIs) selected according to our hypothesis, as described below. For the localization task instead, we entered the contrasted image for the positive effect of S_Loc_ for manipulation check (i.e., A1 activations) and those testing the selective activation of V1 during S_Bis_ (S_Bis_ > S_Loc_) as a dependent variable in a one sample *t* test. We used cluster-extent based thresholding for multiple comparisons correction of statistical maps (pFWE<0.05).

##### ROIs selection

We defined the ROIs for the univariate fMRI analyses by using the Jülich histological (cyto- and myelo-architectonic) atlas.^44^ V1 and A1 corresponded to the Brodmann area 17^45^ and 41,^46^ respectively, divided into left and right sides. We used them as inclusive masks in the fMRI group ROIs analyses separately for each side: specifically, A1 was used in the ANOVA as we were interested in the positive effect of the task, and V1 in the one sample *t* test to explore the activation for the bisection task.

#### Dynamic causal modeling

We used Dynamic Causal Modeling (DCM) to study the changes in effective connectivity between brain regions limiting the analysis to the spatial bisection task, expecting increased effective connectivity from associative brain regions to V1. With this aim, we selected the areas based on our hypotheses showing the recruitment of A1 and of V1 for S_Bis_. In addition, the fMRI results revealed the involvement of the bilateral IPS during the processing of sounds, regardless of the task. Considering our finding and the relevance of the IPS in space processing,^18^^,^^47^ we decided to include the IPS in the DCM analysis.

In the DCM analysis, we used only sequences with S_Bis_. Specifically, for each participant we extracted the time series of each region using the contrast image of the positive effect of S_Bis_ (i.e., S_Bis_ > 0) and considered a sphere (4 mm radius) around the peak of activation. Each contrast was adjusted for the “effect of interest” (F-contrast) to regress out any confounding factors (e.g., head motion) from the timeseries. To reduce noise, only voxels that exceed some liberal statistical threshold for each contrast of interest were retained^48^: more precisely, we selected the clusters in the different brain areas by defining those with at least 10 voxels and set the p uncorrected to be lower than 0.001 at peak level. However, if no voxels were significant, we further decreased the threshold to 0.01 and then 0.05. Table S3 in the Supplementary Materials shows the coordinates (in mm), the dimensions of each cluster and the *p* value for each participant.

We designed the model space with four DCMs, as depicted in Figure 2. We assumed that each model was left-right symmetrical to limit the number of possible models.^49^ All of them included the three brain regions bidirectionally connected. The input to A1 was provided by S_Bis_. The DCMs were implemented in SPM12 as fixed-effect Bayesian model selection for 4 models. The four DCMs allowed us to investigate changes in effective connectivity, due to the bisection task expecting a modulatory effect on both backward and forward connections between low-level sensory regions and IPS. We constructed the four DCMs for each participant and then estimated for each of them three parameters: *driving input*, changes in neural response due to S_Bis_; *modulatory effect of task*, changes in the effective connectivity due to S_Bis_; *baseline connectivity*, changes in neural response due to neural activity (task-independent). More precisely, the baseline connectivity represents the inherent or intrinsic coupling between regions of interest and it is considered orthogonal and independent to the driving input. We did not include Ref as regressor in the DCM analyses, since these stimuli do not elicit responses in V1, and it is not possible to study the effect of modulation on a not significant response. In addition, we were interested in the modulatory effect of S_Bis_ on the three node connectivity network: A1, V1 and IPS. To select the best model, we compared them using fixed-effect Bayesian model selection.^50^ Then, we averaged the connectivity parameters of the best model across participants and entered them as dependent variables in one-sample t tests using MATLAB, testing the null hypothesis that each parameter was not different from zero.^51^
