# Supplementary material for: Primary visual cortex BOLD responses to relative localization of sounds at 7T
Source: iScience. 2026 May 25;29(6):116075. doi: 10.1016/j.isci.2026.116075 (PMC13226777; doi:10.1016/j.isci.2026.116075)
Supplement: Document S1. Figures S1–S3 and Tables S1–S3 [file mmc1.pdf]

iScience, Volume *29*

Supplemental information

Primary visual cortex BOLD responses

to relative localization of sounds at 7T

M. Riberto, M.B. Amadeo, A. Inuggi, M. Costagli, C. Campus, M. Gori, and M.C. Morrone

SUPPLEMENTARY MATERIALS

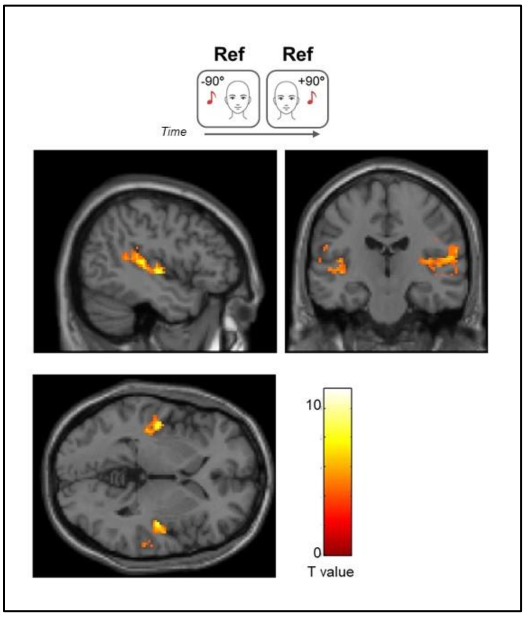

Figure S1. fMRI results. Here we show the whole brain BOLD activations in response to Ref only during the bisection task. Ref elicited significant activations in the bilateral A1 (left: -44 -30 8, k= 862, t= 8.06, pFWE < 0.001; right: 62 -30 14, k= 1314, t= 10.24, pFWE < 0.001), but not in the bilateral V1. The colour bar represents t values associated with each contrast. Abbreviations. Ref, reference landmarks presented at ±90° from the midline. k= number of voxels; A1, primary auditory cortex; V1 primary visual cortex. Related to Figure 1.

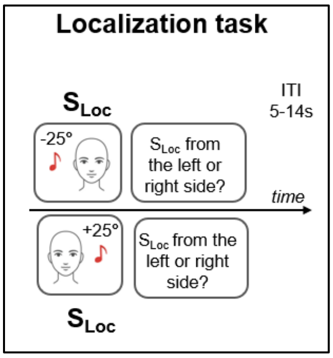

Figure S2. Localization task. During fMRI, after the spatial bisection task, ten participants performed the auditory localization task. Participants' task was to mentally assess whether S_{Loc} placed at ± 25° from midline was coming from either the right or left sides. Abbreviations. ISI, Inter-stimulus interval; ITI, Inter-trial interval. Related to Figure 3.

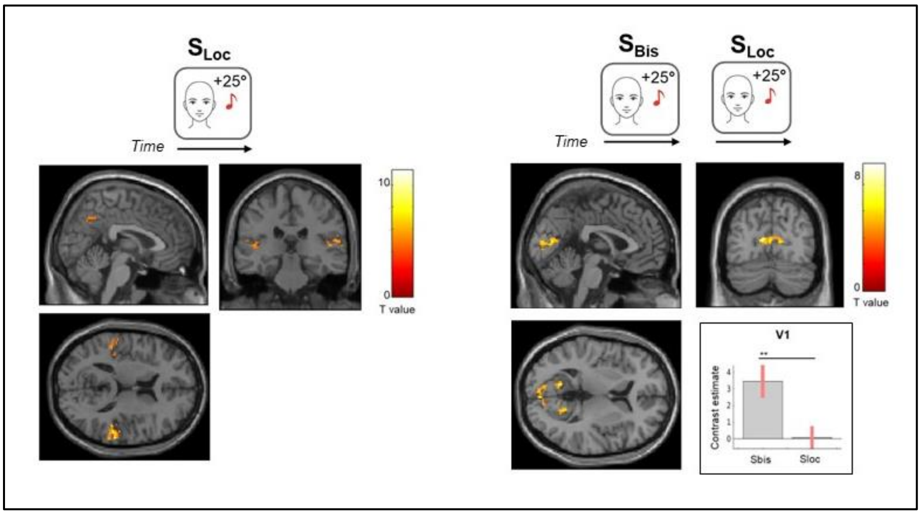

Figure S3. fMRI results. Left. Whole brain activations in the bilateral A1 and right Precuneus in response to S_{Loc}. Right. The recruitment of visual areas was modulated by the type of auditory spatial representation elicited by the task: in S_{Bis} there is significantly increased activation in bilateral V1 that represents the peripheral part of the visual field, and not in auditory cortex. The same pattern of activation was found in the left IPS (not shown in the image). Abbreviations. S_{Bis}, sound presented at ±25° from the midline in the bisection task. S_{Loc}, sound presented at ±25° from the midline in the localization task. Related to Figure 1.

Contrast	Brain region	Coordinates	k	t	pFWE
**S_{Loc}>0**	A1 Right	62 -30 14	221	11.29	<0.001
	A1 Left	-50 -34 14	78	6.53	<0.001
	Precuneus Right	10 -50 38	40	6.16	0.01
**S_{Bis}>S_{Loc}**	V1 Left	-8 -74 14	501	9.56	<0.001
	V1 Right	4 -84 8			
	IPS Left	-34 -46 38	46	6.13	<0.001

Table S1. fMRI results. Coordinates (centre of the cluster), size in voxels (k) and the statistics of brain regions significantly activated in each group analysis. Abbreviations. A1, primary auditory cortex; V1 primary visual cortex; IPL, inferior parietal lobule. Related to Figure 1 and Table 1.

Participant	Age	Sex	Ancestry
1	31	M	European
2	34	F	European
3	26	F	European
4	27	F	European
5	25	F	European
6	26	M	European
7	29	M	Asian
8	24	F	European
9	23	F	European
10	23	M	European

Table S2. Participant demographics. Here we report age, sex, and ancestry for each participant. Related to Table 1.

	T contrast: $S_{Bis} > 0$								
	V1			A1			IPS		
S	x y z	k	p unc	x y z	k	p unc	x y z	k	p unc
1	18 -100 -2	13	<0.001	70 -26 8	4241	<0.001	34 -52 38	1354	<0.001
2	-22 -96 -2	16	<0.001	-46 -12 -10	56	<0.001	-36 -40 36	8953	<0.001
3	-6 -98 4	1532	<0.001	-38 -26 2	19	<0.001	-46 -42 24	20264	<0.001
4	14 -98 -10	15	<0.001	52 -22 8	1606	<0.001	54 -40 30	18	<0.001
5	12 -74 8	10	0.004	54 -30 6	24	<0.001	32 -54 40	31	<0.001
6	-2 -82 -6	749	<0.001	-48 -12 0	27	<0.001	-40 -36 44	25566	<0.001
7	-12 -80 10	23	<0.001	-44 -30 8	30	<0.001	-38 -44 40	33	<0.001
8	-12 -80 10	21	0.001	-40 -18 -18	16	<0.001	-38 -44 42	27145	<0.001
9	0 -86 8	10	<0.001	44 -20 6	21	<0.001	34 -50 38	16	<0.001
10	-16 -102 -8	26	<0.001	-52 -28 6	1552	<0.001	-38 -44 40	16	<0.001

Table S3. Coordinates (centre of the cluster), size in voxels (k) and the statistics of each ROI used in the DCM analysis, for each participant. We selected clusters of at least 10 voxels with p uncorrected < 0.05 at peak level. Clusters in all the ROIs were selected according to the t contrast testing the positive effect of S_{Bis}. Abbreviations. A1, primary auditory cortex; IPS, intra parietal sulcus; V1, primary visual cortex; p unc, p uncorrected. Related to Figure 2.
